# Association of Childhood Adversity With Differential Susceptibility of Transdiagnostic Psychopathology to Environmental Stress in Adulthood

**DOI:** 10.1001/jamanetworkopen.2018.5354

**Published:** 2018-11-30

**Authors:** C. Sophia Albott, Miriam K. Forbes, Justin J. Anker

**Affiliations:** 1 Department of Psychiatry, University of Minnesota Medical School, Minneapolis; 2 Centre for Emotional Health, Macquarie University, Sydney, Australia

## Abstract

**Importance:**

Multivariable comorbidity research indicates that childhood adversity increases the risk for the development of common mental disorders. This risk is explained by underlying internalizing and externalizing transdiagnostic constructs that are amplified by environmental stressors. The differential susceptibility model suggests that this interaction of risk and environment is bidirectional: at-risk individuals will have worse outcomes in high-stress environments but better outcomes in in low-stress environments.

**Objective:**

To test the differential susceptibility model by examining how a history of adverse childhood experiences moderates the association between life stress and transdiagnostic psychopathology.

**Design, Setting, and Participants:**

Data came from the US National Epidemiological Survey on Alcohol and Related Conditions (NESARC), a population-based observational longitudinal survey administered to adults (≥18 years of age). Participants completed the survey at wave 1 (from 2001 through 2002) and wave 2 (from 2004 through 2005). Responses from 34 458 participants were used for the analyses from March 3, 2017, through October 8, 2018.

**Main Outcomes and Measures:**

Latent variables for internalizing-fear, internalizing-distress, externalizing, and general psychopathology were created to represent continuous levels of psychopathology in each wave. Latent variables were also created to represent continuous levels of life stress at each wave. Level of childhood adversity was characterized based on the number of types of childhood adversity experienced (no [0 types], low [1-2 types], and high [≥3 types] exposure). Analyses examined how the interaction between level of childhood adversity and adult life stress was associated with change in adult transdiagnostic psychopathology factors.

**Results:**

Of the 34 458 participants included in the analysis (58.0% women and 42.0% men; mean [SD] age, 46.0 [17.4] years at wave 1 and 49.0 [17.3] years at wave 2), 40.5% had no adverse childhood experiences, 34.6% had 1 to 2, and 24.9% had 3 or more. At wave 1, 61.5% of the sample endorsed at least 1 stressful life event and 27.2% met criteria for at least 1 mental disorder; at wave 2, these figures were 64.7% and 29.7%, respectively. Childhood adversity moderated the association between changes in adult life stress and changes in all transdiagnostic psychopathology factors. Specifically, higher levels of childhood adversity had a stronger association between adult life stress and adult transdiagnostic psychopathology factors. Further, significant differences between childhood adversity groups occurred in the mean scores of all transdiagnostic psychopathology factors for both increases and decreases in life stress, providing preliminary evidence of differential susceptibility.

**Conclusions and Relevance:**

Results provide empirical support for childhood adversity as a differential susceptibility factor engendering heightened functional and dysfunctional reactivity to later stress.

## Key Points

**Question:**

Are adults with a history of childhood adversity more sensitive to both positive and negative effects of environmental stressors?

**Findings:**

In a longitudinal national survey of 34 458 US adults, individuals with high levels of childhood adversity demonstrated higher levels of transdiagnostic psychopathology factors associated with increased life stress and lower levels of transdiagnostic psychopathology factors associated with decreased life stress compared with individuals without childhood adversity. These associations were consistent across all transdiagnostic psychopathology domains.

**Meaning:**

Childhood adversity may represent a differential susceptibility factor making individuals more vulnerable to stressors but also more sensitive to improvements in stressors.

## Teaser

This US national survey examines how a history of adverse childhood experiences moderates the association between life stress and transdiagnostic psychopathology factors in adults responding to the National Epidemiological Survey on Alcohol and Related Conditions.

## Introduction

Exposure to stressful environments is a primary vulnerability factor in the development of psychopathology. The well-established diathesis-stress model^1,2^ has guided much of this research. According to this model, a stressful environment activates a latent diathesis in the form of behavioral, physiological, or genetic predispositions, resulting in the expression of psychopathology.^1,3^ In addition to innate predispositions, a growing body of work indicates that early exposure to adversity engenders lifelong traitlike increases in sensitivity to stressful life events that heighten the risk for psychopathology.^4,5,6^

Childhood exposure to violence and adversity is common. It is estimated that more than one-third of the adult population has been exposed to maltreatment as a child.^7^ A growing literature substantiates the long-term detriment of childhood adversity on adult mental health. For example, exposure to childhood adversity is associated with a higher likelihood of developing a mental disorder in adulthood^8,9^ and greater impairment.^10^ Studies have found that adults with greater exposure to childhood adversity who subsequently experience stressful life events are at elevated risk of developing multiple mental health disorders (major depressive disorder, posttraumatic stress disorder, or substance use disorders) compared with adults with less exposure to childhood adversity.^4,11^ Multivariate comorbidity research has demonstrated that common mental health disorders are instantiations of internalizing and externalizing transdiagnostic constructs.^12^ Childhood adversity has been shown to affect these constructs insofar as childhood adversity is associated with transdiagnostic levels of internalizing (ie, higher levels of mood and anxiety disorders) and externalizing (ie, higher levels of disinhibition and addiction-related disorders) psychopathology.^13,14,15^

Together, these studies highlight the lifelong consequences of childhood adversity. These consequences may reflect changes in physiological systems involved in stress responsivity that have been shown to be altered in adults with experiences of early-life adversity.^16,17,18^ Specifically, early adversity has been hypothesized to lead to changes in endogenous stress response systems (ie, peripheral neuroendocrine, immune, and central neural) that subsequently enhance sensitivity to environmental stressors^19,20^ and, in so doing, increase the risk for the development of mental health disorders. Studies of stress-related epigenetic changes support this hypothesis that early adversity alters the expression of stress hormone factors implicated in the development of stress-related psychopathology.^21,22,23^ For example, Klengel and colleagues^6,24^ showed that childhood trauma-dependent demethylation of a glucocorticoid response element (*FKBP5* [OMIM 602623] codes for a nuclear receptor involved in terminating the stress response to threat) resulted in increased risk for the development of posttraumatic stress disorder in adulthood. Thus, the association between childhood adversity and mental health disorders in adulthood is consistent with increased vulnerability to negative environmental influences in particular.^4,11,25,26^ However, the possibility that adults with a history of childhood adversity may be sensitive to the environment in general—not only to the negative effects of environmental stress—has not been explicitly explored.^27,28^ Specifically, whether this population might also be sensitive to the advantages of positive environments (ie, differentially susceptible) remains unclear.

The differential susceptibility model describes bidirectional sensitivity to high- and low-stress environments based on individual characteristics, including physiological stress responsivity.^19,29,30^ That is, rather than considering individuals as either vulnerable or resilient to the negative effects of stress, the differential susceptibility model regards environmental responsivity along a continuum of greater and lesser plasticity to negatively and positively valenced contexts.^31,32^ To date, research on the long-term effects of childhood adversity has been limited by its focus on negative contexts. Recent animal research has supported the idea that early-life adversity catalyzes differential susceptibility by demonstrating that prenatal stress fosters increased developmental plasticity to environmental influences.^33^ To our knowledge, no population-level studies have examined whether adults with a history of childhood adversity demonstrate heightened sensitivity to improvements in environmental stress. If this is the case, we have a valuable opportunity to improve the quality of life of those most vulnerable to lifelong mental health problems.

The primary aim of the present study was to test whether the dominant diathesis-stress conceptualization of the association between childhood adversity and adulthood psychopathology could be extended to a differential susceptibility framework. The conceptual framework for this study builds on existing diathesis-stress–oriented research^4,11^ in a large, nationally representative sample of adults to examine whether increases and decreases in adult life stress are differentially associated with changes in manifest adult psychopathology as a function of level of childhood adversity.

## Methods

### Sources of Data

Data were drawn from wave 1 (2001 through 2002) and wave 2 (2004 through 2005) of the National Epidemiological Survey on Alcohol and Related Conditions (NESARC). The NESARC was designed and sponsored by the National Institute of Alcohol Abuse and Alcoholism to assess the prevalence of psychiatric disorders among the population of noninstitutionalized civilian adults (≥18 years of age) in the United States, including Alaska, Hawaii, and the District of Columbia. Institutional review board approval was provided by the US Census Bureau and US Office of Management and Budget for wave 1 of the NESARC and by the Westat institutional review board and the Combined Neuroscience Institutional Review Board of the National Institutes of Health for wave 2. Oral informed consent was obtained from all participants in the NESARC. This study followed the Strengthening the Reporting of Observational Studies in Epidemiology (STROBE) reporting guidelines. The institutional review board of the University of Minnesota reviewed the present study and waived the requirement for additional informed consent by the participants.

Sampling involved a multistage probability algorithm to randomly select participants to participate in the survey. For both waves, participants self-identified race/ethnicity, which was coded as white, black, Native American or Alaskan, Asian, Native Hawaiian or Pacific islander, or Hispanic or Latino. The Hispanic origin variable was constructed from an algorithm developed by the US Census Bureau. African American and Hispanic households and adults aged 18 to 24 years were oversampled, and data were adjusted to accommodate oversampling and nonresponse (household and person level). Among those who completed the survey at wave 1 (n = 43 093), 80.4% (n = 34 653) also completed the wave 2 survey a mean (SE) 36 (2.6) months later.^34^

### Sample

Analyses were conducted on the 34 458 participants with data at waves 1 and 2 who also responded to the items used to measure childhood adversity, as described below. Table 1 includes a description of the study sample at both waves. The 195 participants who did not respond to the childhood adversity items represented 0.6% of the participants with longitudinal data and were excluded from the analyses. These participants did not differ substantially from the analytic sample (ie, Cohen *d *< 0.20 or Φ < 0.1) in terms of sex (Φ < 0.001), race/ethnicity (Φ = 0.01), educational attainment (Φ = 0.02), income (Cohen *d* = 0.10), or estimated psychopathology factor scores at wave 1 (Cohen *d* < 0.12), but the other differences were associated with medium effect sizes (ie, Cohen *d* > 0.20 to < 0.50; Φ > 0.1 to < 0.3) and indicated that the excluded participants tended to be older (Cohen *d* = 0.28), have lower estimated factor scores for stress at both waves (wave 1 Cohen *d* = 0.21; wave 2 Cohen *d* = 0.26), and have lower estimated factor scores for psychopathology at wave 2 (Cohen *d* = 0.41-0.43). All other participants were included in all analyses. A small proportion (0%-0.7%) of participants had missing data for 1 or more stressful life event, and these missing data were handled using full-information maximum-likelihood estimation. All psychiatric diagnostic and demographic data were listwise complete. Sampling weights were also used in factor score estimation to limit bias due to nonresponse and attrition from waves 1 to 2.

**Table 1.** Descriptive Statistics for All Variables Included in the Analyses

Characteristic	Data (N = 34 458)	
	Wave 1	Wave 2
Sociodemographic information		
Age, mean (SD), y	46.0 (17.4)	49.0 (17.3)
Female, No. (%)	19 977 (58.0)	NA
White, No. (%)	20 079 (58.3)	NA
Annual personal income, No. (%), $^a^		
≤19 999	16 273 (47.2)	15 096 (43.8)
20 000-34 999	8064 (23.4)	8043 (23.3)
35 000-69 999	7606 (22.1)	8142 (23.6)
≥70 000	2515 (7.3)	3177 (9.2)
Educational level of some college or more, No. (%)	18 869 (54.8)	19 597 (56.9)
Childhood adversity, No. (%)^b^		
Physical abuse	NA	12 213 (35.4)
Sexual abuse	NA	4228 (12.3)
Endangerment	NA	6148 (17.8)
Exposure to domestic violence	NA	5580 (16.2)
Emotional abuse	NA	8174 (23.7)
Neglect	NA	4626 (13.4)
Parental dysfunction		
Serious mental illness	NA	2384 (6.9)
Substance abuse	NA	8122 (23.6)
Incarceration	NA	2476 (7.2)
Psychopathology domain, No. (%)^c^		
Internalizing-distress		
Major depression	2848 (8.3)	3016 (8.8)
Generalized anxiety	816 (2.4)	1361 (3.9)
Dysthymia	814 (2.4)	477 (1.4)
Internalizing-fear		
Panic and agoraphobia	232 (0.7)	303 (0.9)
Social phobia	1000 (2.9)	943 (2.7)
Specific phobia	2595 (7.5)	2755 (8.0)
Externalizing		
Alcohol use disorder	1168 (3.4)	1430 (4.1)
Tobacco use disorder	4004 (11.6)	4506 (13.1)
Marijuana use disorder	99 (0.3)	113 (0.3)
Other drug use disorder	105 (0.3)	153 (0.4)
Antisocial personality disorder	1149 (3.3)	1221 (3.5)
Life stress^c^		
Proximal trauma	980 (2.8)	1892 (5.5)
Major events, No. (%)		
Death of a loved one	11 240 (32.6)	11 613 (33.7)
Being fired or laid off	2136 (6.2)	1885 (5.5)
Long-term unemployment	3013 (8.7)	3166 (9.2)
Getting divorced or separated	2262 (6.6)	1855 (5.4)
Experiencing a financial crisis	4075 (11.8)	4691 (13.6)
Minor events, No. (%)		
Experiencing legal troubles	1917 (5.6)	2687 (7.8)
Changes in housing	5021 (14.6)	7076 (20.5)
Work troubles	2882 (8.4)	2806 (8.1)
Job changes	7463 (21.6)	7203 (20.9)
Relationship problems	1991 (5.8)	2003 (5.8)

Abbreviation: NA, not applicable.

^a^ Variable included in the analyses had 18 income brackets that are summarized herein.

^b^ Analyzed as a count variable categorized into no (0 endorsed childhood adversity categories), low (1-2 childhood adversity categories), and high (≥3 childhood adversity categories) exposures in wave 2.

^c^ Estimated in a latent variable framework described in the Statistical Analysis subsection of the Methods section.

### Measures

#### Psychopathology

Past-year *Diagnostic and Statistical Manual of Mental Disorders* (Fourth Edition) (*DSM-IV*) diagnoses^35^ were assessed using the Alcohol Use Disorders and Associated Disabilities Interview Schedule IV.^36^ Only disorders assessed at both waves were included to examine change in psychopathology across time. The statistical structure of psychopathology in the NESARC is well validated^37,38^ and includes correlated, transdiagnostic internalizing (depression and anxiety disorders) and externalizing (tobacco, alcohol, marijuana, and other drug dependence and lifetime antisocial personality disorder) dimensions.^12^ The internalizing dimension has been further subdivided into the additional dimensions of fear (panic and agoraphobia, social phobia, and specific phobia) and distress (major depression, generalized anxiety, and dysthymia).^12^ Lahey et al^37^ extended this model in the NESARC data to include a general factor of psychopathology to capture the overlap among all common mental disorders.

#### Childhood Adversity

The wave 2 survey contained questions related to exposure to childhood adversity experienced before 18 years of age. Following the methods of McLaughlin et al,^11^ childhood adversity items were organized into the following 9 categories: physical abuse, sexual abuse, endangerment, exposure to domestic violence, emotional abuse, neglect, and parental dysfunction related to serious mental illness, substance abuse, and incarceration. Per McLaughlin et al,^11^ a count variable of the types of childhood adversity experienced was categorized into no (0 endorsed childhood adversity categories), low (1-2 childhood adversity categories), and high (≥3 childhood adversity categories) exposure.

#### Life Stress in Adulthood

Environmental stress was operationalized in line with the methods of McLaughlin et al^11^ based on major and minor life stressors experienced in the past year and proximal traumatic experiences. Specifically, major stressful life events that were assessed at both waves included the death of a loved one, being fired or laid off, long-term unemployment, getting divorced or separated, and experiencing a financial crisis. Minor stressful life events that were assessed at both waves included experiencing legal troubles, changes in housing, work troubles, job changes, and relationship problems. Proximal trauma was assessed as an adulthood posttraumatic stress disorder symptom-inducing event that occurred in the past 3 to 4 years (ie, in the 4 years before wave 1 or between waves 1 and 2).

### Statistical Analysis

Data were analyzed from March 3, 2017, to October 8, 2018. Level of psychopathology was based on estimated internalizing-fear, internalizing-distress, externalizing, and general psychopathology factor scores at each wave. Factor scores were estimated in MPlus (version 7)^39^ using the maximum a posteriori method with complex survey method variables at each wave and the variance-adjusted weighted least squares estimator. Internalizing-fear, internalizing-distress, and externalizing factor scores were generated from a correlated factor model,^38^ and the general psychopathology factor score was derived from a bifactor model.^37^ Level of life stress was estimated in the same latent variable framework (ie, factor scores on a continuous dimension from low to high) based on the shared variance among major and minor stressful life events and proximal trauma. The estimated factor scores indicate individuals’ relative position on the latent variable representing continuous levels of life stress. The latent variable models were constrained to be equal between the 2 waves to ensure the same constructs were being operationalized at both time points, and all latent variables were standardized (ie, with a mean of 0 and a variance of 1), with higher scores indicating higher levels of psychopathology and stress.

The primary analyses were conducted using the mixed procedure in SPSS (version 24; IBM Corporation). Repeated-measures linear mixed-effects modeling was used to examine how change in life stress (ie, mean level and change over time) was associated with changes in each psychopathology domain (ie, internalizing-fear, internalizing-distress, externalizing, and general psychopathology) over time, and whether this association varied as a function of childhood adversity exposure. The steps in Roisman et al^40^ were used to guide the tests for evidence of differential susceptibility, as described below. All analyses controlled for age and sex, as well as basic information regarding race/ethnicity (white [1] or not white [0]), annual personal income ($0 to ≥$100 000), and educational level (no college [0] or some college or more [1]). Sensitivity analyses were also conducted that examined whether sex had a moderation effect in the models in line with recent findings in the wave 1 NESARC data that stressful life events may be more strongly associated with psychopathology for women.^41^ The pattern of the associations did not vary as a function of sex (ie, none of the stress × childhood adversity × sex interactions were significant), so men and women were analyzed together. Significance levels were adjusted for a reportwide false discovery rate of 5% using the Benjamini-Hochberg procedure,^42^ which corresponded with a 2-tailed *P* ≤ .031.

## Results

Among the 34 458 participants included in the analysis (58.0% women and 42.0% men; mean [SD] age, 46.0 [17.4] years at wave 1 and 49.0 [17.3] years at wave 2), 40.5% had no adverse childhood experiences, 34.6% had 1 to 2, and 24.9% had 3 or more. At wave 1, 61.5% of the sample endorsed at least 1 stressful life event and 27.2% met criteria for at least 1 mental disorder; these figures were 64.7% and 29.7%, respectively, in wave 2.

Childhood adversity significantly moderated the association between changes in stress and changes in all transdiagnostic psychopathology domains with remarkably similar effect sizes (Table 2). Specifically, exposure to higher levels of childhood adversity was associated with a stronger association between stress and all transdiagnostic domains of psychopathology (Figure). All interactions were disordinal (or crossover interactions), in line with the differential susceptibility model.^43^ Specifically, the estimated values for the childhood adversity groups had crossover points ranging from −2.29 to −1.73, which were within the observed range of changes in estimated factor scores of stress from wave 1 to wave 2 (range, −3.15 to 3.28).^43^ Further, nonlinearity in the associations could not account for the interactions between childhood adversity and life stress. For example, at the extremities of the distribution of changes in stress between waves, the group with high exposure to childhood adversity had significantly lower estimated levels of psychopathology across all domains, compared to the group with no exposure. The interactions remained significant and largely unchanged in magnitude after including *X*^2^ and *ZX^2^* terms in each of the models (where *X* is life stress and *Z* is childhood adversity). Finally, significant differences occurred between childhood adversity groups in the estimated marginal means for all domains of psychopathology at low and high ends of the distribution of changes in stress over time (Figure). That is, individuals who experienced high levels of childhood adversity had greater changes in psychopathology corresponding with increases and decreases in life stress, providing evidence of differential susceptibility.^40^ However, the proportion of the sample affected by positive changes in the environment at the extremities of the distribution of stress—beyond the crossover points—was consistently small (0.1%-1.2%), as was the proportion of variance accounted for by the terms in each model based on the pseudo-*R*^2^ measure of ω^2^ (0.3%-0.5%).^44^

**Table 2.** Estimated Fixed Effects of Change in Each Transdiagnostic Psychopathology Domain^a^

Construct	Transdiagnostic Psychopathology Domain, Estimated Fixed Effects (95% CI)^b^			
	Internalizing		Externalizing^c^	General Psychopathology^c^
	Fear^c^	Distress^c^		
Intercept	0.29 (1.28 to 0.31)	0.33 (0.31 to 0.34)	0.34 (0.33 to 0.36)	0.33 (0.32 to 0.34)
Life stress	0.23 (0.21 to 0.24)	0.27 (0.26 to 0.28)	0.23 (0.22 to 0.24)	0.26 (0.25 to 0.28)
**Childhood Adversity**				
No vs high exposure	–0.20 (–0.21 to –0.18)	–0.21 (–0.22 to –0.20)	–0.21 (–0.22 to –0.20)	–0.22 (–0.23 to –0.21)
Low vs high exposure	–0.12 (–0.14 to –0.11)	–0.13 (–0.14 to –0.12)	–0.13 (–0.14 to –0.11)	–0.14 (–0.15 to –0.12)
**Stress by Childhood Adversity**				
No vs high exposure	–0.10 (–0.11 to –0.08)	–0.12 (–0.14 to –0.11)	–0.11 (–0.12 to –0.09)	–0.12 (–0.14 to –0.10)
Low vs high exposure	–0.05 (–0.07 to –0.04)	–0.07 (–0.08 to –0.05)	–0.06 (–0.07 to –0.04)	–0.06 (–0.08 to –0.05)

^a^ Includes 34 458 participants. These values represent the estimated associations between each indicator and dependent variable after controlling for age, sex, race/ethnicity minority status, annual personal income, and educational level. Life stress (an indicator) and psychopathology (the dependent variables) are based on estimated factor scores derived from standardized latent variables (ie, with a mean of 0 and a variance of 1).

^b^ Each transdiagnostic psychopathology domain was analyzed separately.

^c^ All of the type III tests of fixed effects and the estimates of the fixed effect (ie, the global *F* test for the effect and the 2-tailed *t* test for the different levels of categorical effects) reached significance after adjusting for a reportwide false discovery rate of 5% using the Benjamini-Hochberg procedure,^42^ which corresponded to *P* ≤ .031.

**Figure.**
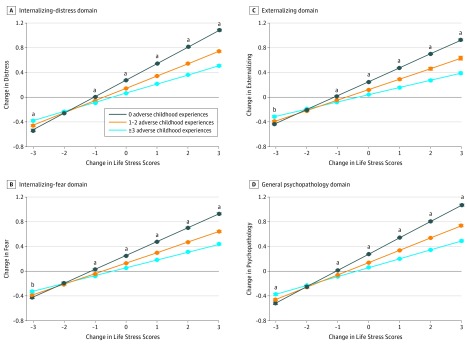
Change in Each Transdiagnostic Psychopathology Domain by Change in Life Stress and Level of Childhood Adversity Changes in each transdiagnostic psychopathology domain and life stress were measured based on estimated factor scores at waves 1 and 2 of the National Epidemiological Survey on Alcohol and Related Conditions (NESARC), derived from standardized latent variables (ie, with a mean of 0 and a variance of 1). The number of individuals corresponding to the different values of observed change scores in life stress was 158 (0.4%) with less than −2, 2504 (7.3%) with −2 to less than −1, 10 236 (29.7%) with −1 to less than 0, 7563 (21.9%) with 0, 10 872 (31.6%) with more than 0 to 1, 2865 (8.3%) with more than 1 to 2, and 260 (0.8%) with more than 2. All analyses were controlled for age, sex, race/ethnicity minority status, annual personal income, and educational level. Error bars depict standard errors for the estimated marginal means. Significance levels were adjusted for a reportwide false discovery rate of 5% using the Benjamini-Hochberg procedure,^42^ which corresponded to *P* ≤ .031. ^a^Indicates *P* ≤ .031 between all adverse childhood experience groups. ^b^Indicates *P* ≤ .031 between participants with no childhood adversity and those with 1 to 2 and 3 or more adverse childhood experiences, but not between the groups with 1 to 2 and 3 or more adverse childhood experiences.

## Discussion

This project examined whether childhood adversity may shape mental health via environmental sensitivity in adulthood. Three novel findings emerged from this study.

First, taken together, these results suggest that childhood adversity may be a differential susceptibility factor. Experiences of childhood adversity were not only associated with psychopathology risk in response to increases in life stress but were also associated with heightened sensitivity to positive changes in the environment (ie, decreased life stress). Specifically, adults with high levels of childhood adversity benefited more from large positive changes in their environment—evident in larger decreases in transdiagnostic psychopathology domains—compared with adults with lower or no exposure to childhood adversity.

Second, the evidence of differential susceptibility was found for all transdiagnostic psychopathology domains. The similar effect sizes for the internalizing-fear, internalizing-distress, and externalizing factors, as well as for the general psychopathology factor, suggest that childhood adversity has broadband transdiagnostic associations across common mental disorders. This finding extends the results of previous studies of these data^4,11^ to a greater range of disorders. Furthermore, by examining change in environmental stress and its association with change in transdiagnostic psychopathology domains, the present study was able to examine the role of childhood adversity in a differential susceptibility framework.

Third, the present results provide the first evidence, to our knowledge, of a transdiagnostic differential susceptibility phenotype at the population level. This finding stands in contrast to those of other studies that have identified differential susceptibility effects via genetic moderation.^45,46,47,48^ Multiple studies of endogenous mechanisms catalyzing stress sensitivity have been reported.^49,50,51,52^ These mechanisms include epigenetic modifications regulating the expression of stress-associated genes. For example, Klengel and Binder^24^ showed how methylation of a functional glucocorticoid response element (*FKBP5*) in adults with a history of child abuse increased the risk for posttraumatic stress disorder after trauma exposure in adulthood. Other studies have reported similar results for multiple genes,^53,54,55,56^ which may represent molecular mechanisms facilitating the development of a differential susceptibility phenotype. These studies, together with the current population-level results, suggest that multiple mechanisms likely lead to the phenotype of heightened stress reactivity catalyzed by childhood adversity. Correspondingly, stratification of the current results according to genotype or physiological stress reactivity may yield larger phenotypic effects than those observed in the present study.

Finding evidence of differential susceptibility at the population level in adults is an important extension of the differential susceptibility model that is implied but has never been empirically tested. Further, extending the diathesis-stress literature on adult psychopathology and childhood adversity into a differential susceptibility framework has important implications for the conceptualization of interventions targeting psychiatric disorders. Existing literature has pointed to the insufficiency of current interventions to appreciably modify the course of most psychiatric disorders.^57,58^ Although the effect sizes in this study are small, the results suggest that improvements in multiple environmental stressors disproportionately benefited individuals with high levels of childhood adversity. Given that these benefits were only observed when substantial reductions in life stress occurred, singular psychosocial interventions (ie, the initiation of a medication therapy or a course of a single evidence-based psychotherapy) may be insufficient for elucidating this effect in this population. To make progress toward identifying psychosocial domains that represent efficient targets for intervention, future research might take a more fine-grained approach toward characterization of the environmental improvements—including supportive and resilience factors—that are associated with reductions of broadband psychiatric symptoms in individuals with high levels of childhood adversity.

Importantly, this study moves beyond the focus of the diathesis-stress literature on the amplified psychopathologic vulnerability and impairment of adults with a history of childhood adversity.^4,11,59,60^ Although the diathesis-stress literature makes a strong argument for early-life interventions aimed at preventing childhood maltreatment, it suggests only poor outcomes for the treatment of adults who have already had these experiences. Contrary to this pessimistic conclusion, this study found that individuals with high levels of childhood adversity may benefit the most from environmental enrichment and shifts the focus toward finding interventions to benefit these individuals when the time for preventive interventions has passed.

### Limitations

Although the use of a large, longitudinal, and nationally representative sample was a key strength of this study, characteristics of the NESARC data also represented the primary limitations. For example, measurement of childhood adversity was based on retrospective reports, which have been found to be biased^61^ and more closely associated with negative life outcomes in adulthood than prospective reports.^62^ Ideally, future research would incorporate prospective or objective measurement of childhood adversity, which would help to determine how bias in retrospective reports may have affected the present results.

In testing for evidence of differential susceptibility, Roisman et al^40^ emphasized the importance not only of establishing a disordinal interaction (ie, a crossover in the groups’ estimated outcomes within the measured range of values)^29,63^ but also of quantifying the size of differential susceptibility associations. Notably, the differential susceptibility associations found in the present study were small. This finding was likely due in part to characteristics of the NESARC data that might suppress evidence of differential susceptibility. For example, our moderator (childhood adversity) is robustly associated with our outcome variable (transdiagnostic psychopathology domains),^13,14,15^ making it more likely that an interaction will appear to be ordinal (ie, interpreted as diathesis-stress only).^40^ In their proposed test for differential susceptibility, Belsky et al^63^ required that moderators and outcome variables should be uncorrelated. However, Roisman et al^40^ suggested that this criterion is unnecessarily conservative because the interaction term that tests for differential susceptibility is a unique set of the associations among the independent variable, moderator, and outcome variable.^40^

As described earlier, we used the steps indicated by Roisman et al^40^ to test for evidence of differential susceptibility, which were readily applied to a longitudinal mixed-modeling analytic framework. Belsky and colleagues^43,64,65^ have proposed an alternative confirmatory framework for testing competing hypotheses regarding evidence of diathesis-stress vs varying levels of evidence of differential susceptibility. This approach would be ideal for future research seeking to replicate or extend these findings because it provides explicit guidelines for quantifying the strength of the evidence of a differential susceptibility effect.

The measures in NESARC were focused on quantifying negative environmental factors and manifest psychopathology (in the form of psychiatric diagnoses) rather than capturing a full range of environmental inputs and outputs, including beneficial environmental factors and mental well-being. Our focus on change over time in these domains allowed us to operationalize a wide spectrum of improvements as well as deterioration in the quality of the environment (and corresponding transdiagnostic psychopathology domains). However, the life stress and psychiatric diagnosis variables did not specifically capture environments and outcomes representative of thriving, further masking evidence of differential susceptibility.^66^ In sum, finding evidence of differential susceptibility despite these characteristics of the study suggests that future research (with a greater spectrum of supportive environments and assessments of mental well-being) may find more robust evidence for differential susceptibility.

## Conclusions

The findings of this study contribute to our understanding of the enduring effects of childhood adversity. The results extend the diathesis-stress model by providing empirical support for the hypothesis that childhood adversity may represent a plasticity factor—rather than only increasing the vulnerability of individuals to stressful life events. Further research is needed to replicate this finding, and subsequently to understand whether psychiatric outcomes are improved by interventions focused on reducing environmental stressors or by interventions that modify the endogenous processes^67^ that underlie heightened reactivity to the environment.
