# Transposed Information in Figure Key

**DOI:** 10.1001/jamanetworkopen.2019.0153

**Published:** 2019-02-15

In the Original Investigation titled “Association of Childhood Adversity With Differential Susceptibility of Transdiagnostic Psychopathology to Environmental Stress in Adulthood,”^1^ the colors in the figure’s key indicating “0 adverse childhood experiences” and “≥3 adverse childhood experiences” were transposed. The cyan line should have indicated “0 adverse childhood experiences” and the navy line should have indicated “≥3 adverse childhood experiences.” This article has been corrected.^1^
